# In ‘real world’ patients with COPD, exacerbation history, and not blood eosinophils, is the most reliable predictor of future exacerbations

**DOI:** 10.1186/s12931-023-02311-x

**Published:** 2023-01-05

**Authors:** Heinrich Worth, Roland Buhl, Carl-Peter Criée, Peter Kardos, Eva Gückel, Claus F. Vogelmeier

**Affiliations:** 1 Facharztforum Fürth, 90762 Fürth, Germany; 2 Pulmonary Department, Mainz University Hospital, 55131 Mainz, Germany; 3 Department of Sleep and Respiratory Medicine, Evangelical Hospital Goettingen-Weende, 37120 Bovenden, Germany; 4 Group Practice and Centre for Allergy, Respiratory and Sleep Medicine, Red Cross Maingau Hospital, 60316 Frankfurt am Main, Germany; 5 Clinical Research, Respiratory, Novartis Pharma GmbH, 90429 Nürnberg, Germany; 6 Department of Medicine, Pulmonary and Critical Care Medicine, German Center for Lung Research (DZL), University Medical Centre Giessen and Marburg, Philipps-University Marburg, 35043 Marburg, Germany

## Abstract

**Introduction:**

There is an interest in the role of blood eosinophils for predicting inhaled corticosteroid (ICS) response in chronic obstructive pulmonary disease (COPD). Most data are from interventional clinical studies; data from unselected real-world populations may help better inform treatment decisions. DACCORD is a non-interventional real-world study. Cohort 3 recruited patients with COPD who had received triple therapy for ≥ 6 months; prior to entry patients either continued triple therapy, or switched to a long-acting muscarinic antagonist/long-acting beta_2_-agonist (LABA/LAMA), and were followed for 12 months.

**Methods:**

For these *post-hoc* analyses, patients were divided into four groups based on exacerbation history and baseline blood eosinophil count (< 100 vs. > 300 cells/µL). Exacerbation rates were calculated overall and for the two treatments.

**Results:**

Among the 430 patients in the current analyses, the largest groups had low exacerbation history with high (44.2%) or low eosinophils (36.7%). Most patients did not exacerbate during follow-up (68.8% overall; 83.2% and 63.7% with LABA/LAMA and triple therapy). The highest exacerbation rates were in groups with high exacerbation history, differing significantly in the overall analyses from those with low exacerbation history (matched by eosinophil count); rates did not differ when grouped by eosinophil count (matched by exacerbation history).

**Conclusions:**

Although most patients in these analyses did not exacerbate during follow-up, whereas exacerbation history is a predictor of future exacerbations, blood eosinophil count is not. This suggests that although eosinophil count may help to guide ICS initiation, this is less of a consideration when ‘stepping-down’ from triple therapy to a LABA/LAMA

## Introduction

There is an ongoing debate about potential clinical and laboratory parameters that may help identify the patients with chronic obstructive pulmonary disease (COPD) who may benefit from inhaled triple therapy, and those in whom triple therapy may be ‘stepped-down’ to a long-acting muscarinic antagonist plus long-acting beta2-agonist combination (LABA/LAMA). Some analyses have suggested that blood eosinophils can guide treatment choice and/or predict treatment response in patients with COPD [1, 2], and blood eosinophil levels are recommended in the Global Initiative for Chronic Obstructive Lung Disease (GOLD) strategy document as one of the prime factors to consider when initiating inhaled corticosteroid (ICS) treatment [3]. However, whereas in asthma blood eosinophil levels are clearly associated with subsequent exacerbation risk [4–6], results in COPD are mixed, and the GOLD Scientific Committee has advised that blood eosinophil levels cannot be used as a standalone biomarker of future risk [7]. Consistent with this, in a pooled analysis of data from 11 studies there was no clinically important relationship between baseline blood eosinophil count and exacerbation rate [8]. Similarly, in a prospective observational study there was no relationship between blood eosinophil count and subsequent prognosis [9]. However, these analyses did not take into account the relationship between eosinophil levels and medication usage (specifically ICS use) on exacerbation risk. Indeed, in a pooled analysis of interventional clinical studies, there was a complex association of prior and current medication, exacerbation history, and baseline eosinophil count with subsequent exacerbation risk – with baseline eosinophil count correlating with subsequent exacerbation risk in those who had ICS withdrawn [10]. A major confounding factor in such interventional studies are their inclusion criteria, with almost all recruiting patients at high exacerbation risk, therefore limiting the validity of these analyses. Similar analyses in unselected real-world populations may therefore help better inform treatment decisions.

DACCORD is a longitudinal, non-interventional real-world study that has recruited patients with COPD in three cohorts [11–17]. The third cohort, which is the subject of this manuscript, recruited patients who had been receiving triple therapy for at least six months; the treating physician then either continued triple therapy, or switched the patient to a LABA/LAMA fixed-dose combination (FDC) prior to entry, with these patients followed up for 12 months [17]. Following this ‘step-down’ there was no overall worsening in COPD – indeed, some patients had better outcomes after being switched to LABA/LAMA FDC than those continuing triple therapy, with a lower proportion of patients in the LABA/LAMA group having COPD worsening or exacerbating [17].

One of the prespecified analyses for this cohort was of the predictive value of blood eosinophil values (assessed up to six months prior to entry) for subsequent events, including exacerbations [17]. There was no consistent relationship between eosinophil values and exacerbations, in that patients with the highest baseline eosinophil values in both groups did not exacerbate during the study, whereas patients with the highest number of exacerbations had low eosinophil counts. However, the prespecified analyses did not take exacerbation history into account, and we therefore decided to perform additional post-hoc analyses of these data, combining eosinophil counts with exacerbation history.

## Methods

### Trial design

Patients in Cohort 3 of DACCORD were recruited at 85 primary and secondary care practices distributed throughout Germany. Eligible patients were adults aged at least 40 years who had a confirmed diagnosis of COPD, were included in the Disease Management Program (DMP) for COPD or fulfilled the criteria for inclusion, had been receiving LABA + LAMA + ICS therapy for at least six months prior to baseline, had available data on blood eosinophil count (determined within six months prior to inclusion), and provided written informed consent. Exclusion criteria were limited to inclusion in the asthma DMP, concomitant asthma or a prior asthma diagnosis, foreseeable problems in follow-up across the study duration, or current randomised controlled trial participation.

Prior to study entry, each patient’s physician decided, in agreement with the patient, to either continue maintenance treatment with triple therapy, or switch to a LABA/LAMA FDC. Given the non-interventional ‘real-life’ nature of the study, this decision was to have been made by the treating physician based only on the individual circumstances of the patient, and was neither influenced by the fact that patients were included in DACCORD nor by the protocol itself. Specific visits were not mandated by the protocol, but, consistent with usual care in Germany, it was anticipated that data would be recorded approximately every three months.

The study was registered in the European Network of Centers for Pharmacoepidemiology and Pharmacovigilance (EUPAS4207; http://www.encepp.eu/encepp/viewResource.htm?id=6316) and was approved by the ethics committee of the University of Erlangen-Nürnberg, Germany.

### Statistical methods

For these *post-hoc* analyses, patients were grouped as follows:

- Group 1 (low exacerbation history and high baseline eosinophils): 0–1 non-hospitalised and 0 hospitalised exacerbations in the year prior to entry, and > 300 eosinophils/µL at baseline;
- Group 2 (high exacerbation history and high baseline eosinophils): ≥2 non-hospitalised or ≥ 1 hospitalised exacerbations in the year prior to entry, and > 300 eosinophils/µL at baseline;
- Group 3 (low exacerbation history and low baseline eosinophils): 0–1 non-hospitalised and 0 hospitalised exacerbations in the year prior to entry, and < 100 eosinophils/µL at baseline;
- Group 4 (high exacerbation history and low baseline eosinophils): ≥2 non-hospitalised or ≥ 1 hospitalised exacerbations in the year prior to entry, and < 100 eosinophils/µL at baseline.

The exacerbation rate was estimated within these groups, both overall and for the two treatments, using a negative binomial regression model with annualised numbers of exacerbation as dependent variable and no independent variable. Data were analysed in recruited patients who completed the end-of-study visit and at least two of the three intermediate visits, and who had no relevant deviations from the observational plan. In order to create a clear distinction between the low and high eosinophil groups, patients with between 100 and 300 eosinophils/µL at baseline were excluded from these analyses.

## Results

**Fig. 1:**
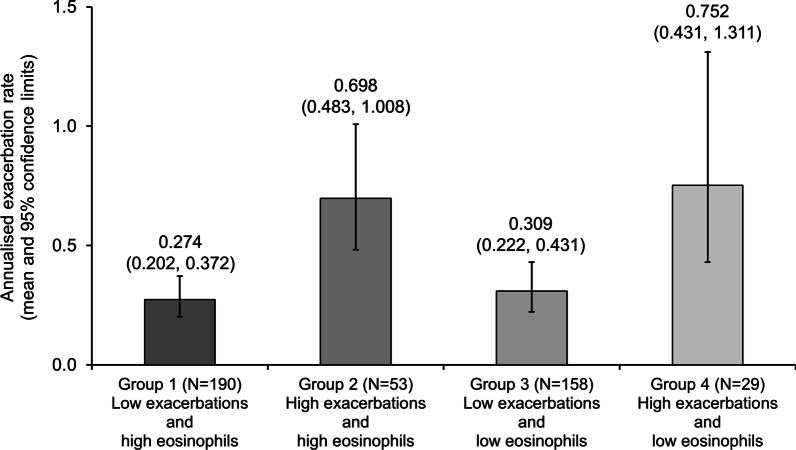
Annualised exacerbation rates during the follow-up period, with patients categorised by exacerbation history and eosinophil count in the overall population. Group 1: 0–1 non-hospitalised and 0 hospitalised exacerbations in the year prior to entry and > 300 eosinophils/µL at baseline; Group 2: ≥2 non-hospitalised or ≥ 1 hospitalised exacerbations in the year prior to entry and > 300 eosinophils/µL at baseline; Group 3: 0–1 non-hospitalised and 0 hospitalised exacerbations in the year prior to entry and < 100 eosinophils/µL at baseline; Group 4: ≥2 non-hospitalised or ≥ 1 hospitalised exacerbations in the year prior to entry and < 100 eosinophils/µL at baseline

Cohort 3 of DACCORD ran between January 2018 and January 2021. Of the 1192 patients recruited, 967 had valid baseline data and completed at least two of the three quarterly visits with no major protocol deviations [17]. After excluding those with baseline eosinophil count between 100 and 300 cells/µL the current analyses include data from 430 patients. In the overall population, the largest group of patients was Group 1 (low exacerbation history and high baseline eosinophils [44.2%]), followed by Group 3 (low exacerbation history and low baseline eosinophils [36.7%]; Table 1). Most of the patients (68.8%) did not exacerbate during the follow-up period. The highest proportions of patients who did exacerbate were in Groups 2 and 4 (the groups with a high exacerbation history), although patients who exacerbated still comprised less than 50% of each group. These data were consistent with the exacerbation rate data, in that the rates in all four groups were low, but the highest rates were in the two groups with a high exacerbation history (Fig. 1). Furthermore, as indicated by the 95% confidence intervals, the exacerbation rates in Groups 1 and 3 (low exacerbation history) differed significantly from those of Groups 2 and 4 (high exacerbation history) when matched by baseline eosinophil category. However, the exacerbation rates in Groups 1 and 2 (high baseline eosinophil count) did not differ from those in Groups 3 and 4 (low eosinophil count) when matched by exacerbation history. Patients recruited into DACCORD Cohort 3 had predominantly mild to moderate airflow limitation (assessed at standard clinic visits without requiring washout of maintenance therapy and not necessarily post-bronchodilator), with mean (SD) forced expiratory volumes in 1 s of 66.9% (24.7) and 57.7% (22.8) in the LABA/LAMA FDC and triple therapy groups, respectively.

**Table 1:** Proportion of patients exacerbating during the follow-up period, with patients categorised by exacerbation history and eosinophil count

Patients (%)	Group 1 Low exacerbations and high eosinophils	Group 2 High exacerbations and high eosinophils	Group 3 Low exacerbations and low eosinophils	Group 4 High exacerbations and low eosinophils	Total
Overall population	(N = 190)	(N = 53)	(N = 158)	(N = 29)	(N = 430)
No exacerbation	137 (72.1)	27 (50.9)	117 (74.1)	15 (51.7)	296 (68.8)
One or more exacerbation	41 (21.6)	26 (49.1)	35 (22.2)	13 (44.8)	115 (26.7)
Missing values	12 (6.3)	0	6 (3.8)	1 (3.4)	19 (4.4)
LABA/LAMA fixed–dose combination group	(N = 50)	(N = 4)	(N = 57)	(N = 2)	(N = 113)
No exacerbation	43 (86.0)	2 (50.0)	48 (84.2)	1 (50.0)	94 (83.2)
One or more exacerbation	5 (10.0)	2 (50.0)	9 (15.8)	1 (50.0)	17 (15.0)
Missing values	2 (4.0)	0	0	0	2 (1.8)
Triple therapy	(N = 140)	(N = 49)	(N = 101)	(N = 27)	(N = 317)
No exacerbation	94 (67.1)	25 (51.0)	69 (68.3)	14 (51.9)	202 (63.7)
One or more exacerbation	36 (25.7)	24 (49.0)	26 (25.7)	12 (44.4)	98 (30.9)
Missing values	10 (7.1)	0	6 (5.9)	1 (3.7)	17 (5.4)

Group 1: 0-1 non-hospitalised and 0 hospitalised exacerbations in the year prior to entry and >300 eosinophils/μL at baseline; Group 2: ≥2 non-hospitalised or ≥1 hospitalised exacerbations in the year prior to entry and >300 eosinophils/μL at baseline; Group 3: 0-1 non-hospitalised and 0 hospitalised exacerbations in the year prior to entry and <100 eosinophils/μL at baseline; Group 4: ≥2 non-hospitalised or ≥1 hospitalised exacerbations in the year prior to entry and <100 eosinophils/μL at baseline

Fewer patients entered DACCORD receiving LABA/LAMA FDC than triple therapy (340 [30.2%] vs. 784 [69.8%] in the full DACCORD population). For the current analyses, the proportion of patients receiving the two therapies was consistent with the full DACCORD population (26.3% vs. 73.7%), resulting in very low patient numbers in some of the groups (LABA/LAMA FDC Groups 2 and 4 contained only 4 and 2 patients, respectively). Despite this, the pattern of exacerbations was similar when analysed by treatment to that in the overall population, with the majority of patients not exacerbating (83.2% receiving LABA/LAMA FDC and 63.7% receiving triple therapy), and the highest proportions of patients who did exacerbate being in Groups 2 and 4 (i.e., the high exacerbation history groups), although patients who exacerbated still comprised approximately 50% of each group (Table 1).

**Fig. 2:**
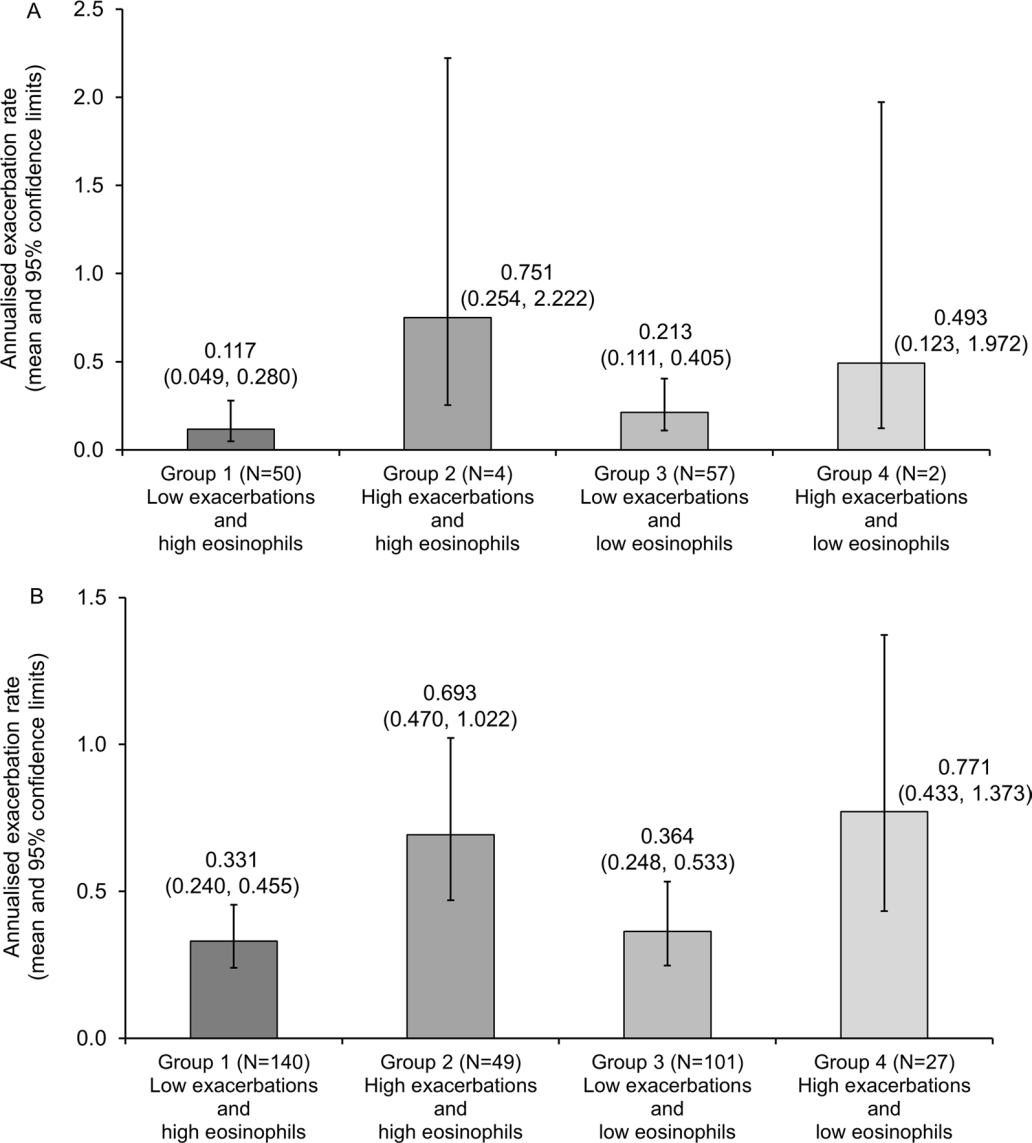
Annualised exacerbation rates during the follow-up period, with patients categorised by exacerbation history and eosinophil count: **A** in the LABA/LAMA fixed-dose combination group; **B** in the triple therapy group. Group 1: 0–1 non-hospitalised and 0 hospitalised exacerbations in the year prior to entry and > 300 eosinophils/µL at baseline; Group 2: ≥2 non-hospitalised or ≥ 1 hospitalised exacerbations in the year prior to entry and > 300 eosinophils/µL at baseline; Group 3: 0–1 non-hospitalised and 0 hospitalised exacerbations in the year prior to entry and < 100 eosinophils/µL at baseline; Group 4: ≥2 non-hospitalised or ≥ 1 hospitalised exacerbations in the year prior to entry and < 100 eosinophils/µL at baseline

The exacerbation rate pattern in the two treatment groups was also consistent with the overall population, although the lower patient numbers resulted in wide and overlapping confidence intervals (Fig. 2). In all four of the analysed groups, exacerbation rates were numerically higher in those receiving triple therapy than in those receiving LABA/LAMA FDC.

## Discussion

Exacerbation history is recognised as a key predictor of future exacerbation risk, with results from other non-interventional studies such as ECLIPSE [18] helping to inform treatment recommendations [3]. The current analyses, using data collected at standard clinic visits from patients who were receiving triple therapy for at least six months prior to entry and who then either continued triple therapy or switched to a LABA/LAMA FDC prior to entry, support and extend these previous findings. In the overall population there was a clear separation between those individuals with a low exacerbation history (defined consistently with GOLD) and those with a high exacerbation history. Just under three quarters of patients with a low exacerbation history did not experience any exacerbations during the follow-up period, compared with approximately half of those with a high exacerbation history, and with significantly lower mean exacerbation rates in the low exacerbation history groups than the high exacerbation history groups when matched by baseline eosinophil category.

Although when analysed by treatment the low patient numbers resulted in high variability around the mean exacerbation rate, the same trend was seen with both treatments. It is of note that despite ICS treatment being discontinued prior to inclusion, the mean exacerbation rates over the follow-up period were all lower in those receiving a LABA/LAMA FDC than in those receiving triple therapy, with approximately 85% of patients receiving a LABA/LAMA FDC and who had a low exacerbation history not exacerbating during the follow-up period, suggesting that the treating physicians were able to select patients who could be ‘stepped down’ to a LABA/LAMA.

Baseline blood eosinophil count, however, did not correlate with subsequent exacerbations, either in the overall analysis or with either treatment. These results contrast somewhat with those of a previous analysis of pooled data, in which baseline eosinophil count correlated with subsequent exacerbation risk in those who had ICS withdrawn [10] (so the equivalent of those receiving a LABA/LAMA FDC in the current analyses, given these patients were previously receiving ICS + LABA + LAMA triple therapy). However, this previous analysis used data from a series of interventional clinical studies that recruited patients with a much higher exacerbation risk than those recruited into DACCORD, and who are much more likely to reflect patients with COPD in the ‘real world’ – 65% in the pooled analyses had a history of at least one moderate/severe exacerbation compared to 45% in the overall DACCORD population [17]. This difference clearly highlights that outcomes of clinical studies in highly selected patient populations cannot always be extrapolated to a real-world population, and emphasises the importance of conducting studies in patients who are as close to the ‘real life’ patient as possible.

The main limitations of these analyses are, of course, also associated with this key strength – the purely non-interventional nature of the study. The only data available are those collected from standard clinic visits, and so it would be unrealistic to recruit only patients who had a blood eosinophil count conducted just prior to study entry. We therefore selected a period of six months to collect these data – although 92.2% had values assessed within three months of entry [17]. Secondly, given the non-interventional nature of the study, the decision to switch the patient from triple to LABA/LAMA FDC therapy (or maintain triple therapy) had to be taken prior to inclusion into the study, with the protocol not impacting treatment choice, and thus patients could not be randomised to therapy, resulting in very low numbers of patients in some subgroups. In addition, all data were collected from the study centres’ own equipment and laboratories. However, these limitations are all consistent with standard care. Furthermore, we do not know why triple therapy was previously initiated in these patients. Finally, the non-interventional nature also meant that medication changes were not prohibited by the protocol, and so it was theoretically possible for all patients who switched to LABA/LAMA FDC to subsequently resume triple therapy. In the event, the majority of patients continued with the same treatment regimen for the duration of follow-up (87.7% and 89.3% of patients in the LABA/LAMA FDC and triple therapy groups respectively) [17].

In conclusion, although the majority of patients included in these analyses did not exacerbate during the follow-up period, whereas exacerbation history is a predictor of future exacerbations, blood eosinophil count is not. This suggests that although eosinophil count may help to guide initiation of ICS therapy (or indeed when not to initiate an ICS) [3], this is less of a consideration when ‘stepping down’ from triple therapy to a LABA/LAMA FDC. Indeed, as was shown in the main analyses, some patients may have a clinical benefit from this step-down [17].

## Data Availability

Anonymised patient data can be requested for further research by submitting a study proposal to www.clinicalstudydatarequest.com.

## Abbreviations

COPD: Chronic obstructive pulmonary disease
DMP: Disease Management Program
FDC: Fixed-dose combination
GOLD: Global Initiative for Chronic Obstructive Lung Disease
ICS: Inhaled corticosteroid
LABA: Long-acting β2-agonist
LAMA: Long-acting muscarinic antagonist

## References

[1] Bafadhel M, Peterson S, De Blas MA, Calverley PM, Rennard SI, Richter K (2018). Predictors of exacerbation risk and response to budesonide in patients with chronic obstructive pulmonary disease: a post-hoc analysis of three randomised trials. Lancet Respir Med.

[2] Singh D, Bafadhel M, Brightling CE, Sciurba FC, Curtis JL, Martinez FJ (2020). Blood eosinophil counts in clinical trials for chronic obstructive pulmonary disease. Am J Respir Crit Care Med..

[3] Global Initiative for Chronic Obstructive Lung Disease. Global strategy for the diagnosis, management, and prevention of chronic obstructive pulmonary disease [Internet]. 2023. https://doi.org/https://goldcopd.org/2023-gold-report-2/. Accessed 15 Nov 2022.

[4] Suruki RY, Daugherty JB, Boudiaf N, Albers FC (2017). The frequency of asthma exacerbations and healthcare utilization in patients with asthma from the UK and USA. BMC Pulm Med.

[5] Malinovschi A, Fonseca JA, Jacinto T, Alving K, Janson C (2013). Exhaled nitric oxide levels and blood eosinophil counts independently associate with wheeze and asthma events in National Health and Nutrition Examination Survey subjects. J Allergy Clin Immunol.

[6] Zeiger RS, Schatz M, Dalal AA, Chen W, Sadikova E, Suruki RY (2017). Blood eosinophil count and outcomes in severe uncontrolled asthma: a prospective study. J Allergy Clin Immunol Pract.

[7] Singh D, Agusti A, Martinez FJ, Papi A, Pavord ID, Wedzicha JA (2022). Blood eosinophils and chronic obstructive pulmonary disease: a global initiative for chronic obstructive lung disease science committee 2022 review. Am J Respir Crit Care Med.

[8] Singh D, Wedzicha JA, Siddiqui S, de la Hoz A, Xue W, Magnussen H (2020). Blood eosinophils as a biomarker of future COPD exacerbation risk: pooled data from 11 clinical trials. Respir Res BioMed Central.

[9] Martínez-Gestoso S, García-Sanz MT, Calvo-Álvarez U, Doval-Oubiña L, Camba-Matos S, Salgado FJ (2021). Variability of blood eosinophil count and prognosis of COPD exacerbations. Ann Med Ann Med.

[10] Singh D, Hurst JR, Martinez FJ, Rabe KF, Bafadhel M, Jenkins M (2022). Predictive modeling of COPD exacerbation rates using baseline risk factors. Ther. Adv Respir Dis Ther Adv Respir Dis.

[11] Kardos P, Vogelmeier C, Buhl R, Criée C-P, Worth H (2015). The prospective non-interventional DACCORD Study in the National COPD Registry in Germany: design and methods. BMC Pulm Med.

[12] Buhl R, Criée C-P, Kardos P, Vogelmeier C, Lossi N, Mailaender C (2016). A year in the life of german patients with COPD: the DACCORD observational study. Int J Chron Obstruct Pulmon Dis.

[13] Vogelmeier C, Worth H, Buhl R, Criée C-P, Lossi NS, Mailänder C (2017). “Real-life” inhaled corticosteroid withdrawal in COPD: a subgroup analysis of DACCORD. Int J Chron Obstruct Pulmon Dis.

[14] Worth H, Buhl R, Criée C-P, Kardos P, Mailänder C, Vogelmeier C (2016). The “real-life” COPD patient in Germany: the DACCORD study. Respir. Med.

[15] Kardos P, Vogelmeier C, Worth H, Buhl R, Lossi NSS, Mailänder C (2017). A two-year evaluation of the ‘real life’ impact of COPD on patients in Germany: the DACCORD observational study. Respir Med.

[16] Worth H, Buhl R, Criée C-P, Kardos P, Lossi NS, Vogelmeier CF (2017). GOLD 2017 treatment pathways in ‘real life’: an analysis of the DACCORD observational study. Respir Med.

[17] Vogelmeier CF, Worth H, Buhl R, Criée C-P, Gückel E, Kardos P (2022). Impact of switching from triple therapy to dual bronchodilation in COPD: the DACCORD ‘real world’ study. Respir Res.

[18] Hurst JR, Vestbo J, Anzueto A, Locantore N, Müllerova H, Tal-Singer R (2010). Susceptibility to exacerbation in chronic obstructive pulmonary disease. N Engl J Med.

